# Development of modular polymeric nanoparticles for drug delivery using amine reactive chemistry

**DOI:** 10.3389/jpps.2024.13148

**Published:** 2024-08-06

**Authors:** Calvin Wong, Emmanuel A. Ho

**Affiliations:** ^1^ School of Pharmacy, University of Waterloo, Waterloo, ON, Canada; ^2^ Waterloo Institute for Nanotechnology, University of Waterloo, Waterloo, ON, Canada

**Keywords:** nanoparticles, curcumin, cancer, drug delivery systems, PLGA

## Abstract

Curcumin has been explored for its anti-cancer potential, but is severely limited by its hydrophobicity and sensitivity to light and water. In this study, poly (lactic-co-glycolic) acid (PLGA) nanoparticles (NPs) were synthesized to encapsulate curcumin via single emulsion method to improve curcumin stability and bioavailability. The PLGA NPs were coated with oligomeric chitosan (COS) and RGD peptide (a peptide consisting of Arg-Gly-Asp) using amine-reactive chemistry (NHS and EDC). Both COS and RGD had been previously shown to accumulate and target many different types of cancer cells. NPs were characterised based on size distribution, zeta potential, and binding efficiency of RGD peptide. They were also evaluated on encapsulation efficiency, and stability, of curcumin within the NPs. OVCAR-3 cancer cells were treated with COS and RGD-coated PLGA NPs loaded with Coumarin-6 dye for fluorescent imaging of cell uptake. They were also treated with curcumin-loaded NPs to determine cytotoxicity and effectiveness of delivery. The NPs exhibited size distribution and zeta potential within expected values, though binding efficiency of RGD was low. Curcumin-loaded NPs showed significant increase in cytotoxicity over free (unencapsulated) curcumin, and void (empty) NPs, suggesting successful delivery of curcumin as an anti-cancer agent; the performance of COS and RGD coated NPs over bare PLGA NPs was inconclusive, however, optimization will be required to improve formulation during the coating steps. This method of NP synthesis serves as proof of concept for a modular solution to the development of various coated polymeric NPs for other drugs or applications.

## Introduction

### Challenges in chemotherapy development

Cancer is one of the leading causes of death worldwide, accounting for nearly 10 million deaths in 2020 [1]. In Canada alone, over 1.5 million individuals have lived or are living with cancer, with over 230,000 new cases and 85,000 deaths in 2022. It is estimated that 43% of people will be diagnosed with cancer in their lifetime [2]. While incidence and death rates have slowly but steadily declined in recent decades, cancer remains a major concern with an aging and growing population.

Chemotherapy is extremely important in the treatment of cancer; in some instances, it is the primary treatment method, particularly with non-solid tumours like leukemia. However, in many instances, chemotherapy is an adjunctive treatment, used with or after surgery or radiation therapy, as patient relapse or metastasis is common [3]. Several factors go into deciding the therapeutic response to a tumour diagnosis. Tumour type is most obvious, as tumour cells have differing sensitivity, responsiveness, or resistance to various therapeutic agents. L-asparaginase, for example, is known to be effective against lymphocytic leukemias, depriving those cells of asparagine, which is essential to their function and survival [4]. Tumour location can pose specific challenges to treatment, such as the difficulty of delivering chemotherapeutic agents to a relatively avascular region, or across the blood-brain-barrier. High doses can systematically force drug to the desired site, but this also risks high systemic toxicity. Smaller tumours tend to be more responsive to treatment than larger tumours; many chemotherapeutic agents work on cells in active growth cycles, and smaller tumours tend to have a larger fraction of cells in this phase [5]. Smaller tumour burden also tends to correlate with healthier patient condition, which often means the patient is able to better tolerate the toxicity of the chemotherapy.

In general, treatment is limited by the negative effects a chemotherapeutic agent has on healthy cells. The ratio of doses between therapeutic effect and toxicity is known as “therapeutic index.” Some adverse effects due to chemotherapy toxicity include gastrointestinal distress, muscular weakness, alopecia, and loss of sensation. However, more severe side effects include cardiac damage, liver damage, and leukopenia/thrombocytopenia, which leaves patients vulnerable to infection or hemorrhaging, respectively [6].

The goal in development of chemotherapy technology is to increase the therapeutic index of both existing and novel treatments and agents. This can be done by increasing the therapeutic effect of an agent, and by decreasing its toxicity. Stronger chemotherapeutic agents have been in development to combat drug-resistant tumours that often lead to treatment failure or patient relapse [3]; they are designed to more aggressively eliminate a larger percentage of tumour cells. Unfortunately, many of these agents do not make it past clinical trials to market, as their increased effectiveness against tumour cells also corresponds with an increased toxicity to healthy cells, leading to more severe systemic toxicity. In addition to toxicity, the development of new chemotherapy drugs is largely limited by bioavailability – the amount of drug in systemic circulation in the body; bioavailability affects the frequency and dosage required for a drug to reach therapeutic concentrations [7]. Hydrophobicity, degradation under physiological conditions or enzymatic processes, and rapid renal clearance are all factors that can negatively impact a drug’s bioavailability and efficacy. Curcumin, derived from the herb, *Curcuma longa* (commonly known as turmeric in the culinary world), has been identified to have anti-cancer potential due to its ability to interact with a wide range of molecular targets, including multiple signalling pathways related to cancer growth and progression [8–12]. However, its use is limited by hydrophobicity, low bioavailability. And rapid decomposition at neutral or alkaline pH, or exposure to light.

One subset of the field of nanomedicine focuses not on the development of novel drugs and other therapeutic agents, but instead on the development of technologies to improve properties of existing chemotherapeutic agents. Nano and microscale structures and systems greatly expand the potential for drug delivery through several potential factors. Depending on the delivery system, they can assist in drug stability and circulatory half-life, enhance the viability of normally low bioavailable drugs, and/or facilitate targeted delivery and uptake [13]. As a result, these systems can increase therapeutic efficacy, reduce toxicological side effects, and improve patient compliance [14]. Polymeric NPs in particular have been explored as attractive nanoscale drug carriers. Encapsulation of drugs into these nanostructures improves stability and effective solubility, and polymeric NPs have been shown to be easily synthesized through both “bottom-up” methods (e.g., nanoprecipitation, salting out) [15, 16] and “top-down” methods (e.g., solvent emulsion) [17].

### Synthetic and natural polymers in NP synthesis

Several synthetic polymers including poly (lactic-*co*-glycolic) acid (PLGA) and poly (ethylene glycol) (PEG) have been approved for use as drug delivery carriers worldwide. PLGA is biocompatible and biodegradable, and its biodegradation and release rate can be controlled by modifying the ratio between the hydrophobic and hydrophilic units (lactic and glycolic acid, respectively) [13, 18]. PLGA nanoparticles encapsulating either hydrophobic or hydrophilic drugs can be easily synthesized using the single or double emulsion method, respectively, and will slowly degrade via hydrolysis to release its load over a period of weeks to months [17].

Additionally, the interest in natural polymers (chitosan, hyaluronic acid, cellulose, etc.) as drug delivery nanomaterial candidates have grown, due to general factors such as biocompatibility, biodegradability, inexpensiveness, and availability [19–21]. Specifically, chitosan has been investigated as a nanocarrier due to the amine groups along its backbone, which make it the only positively charged polysaccharide, and provide reliable points of conjugation for targeting moieties and other structural modifications [22, 23]. A 2014 study showed that chitosan-coated nanoparticles exhibited significant mucoadhesive properties compared to their uncoated counterparts; it is suggested that this property is related to the electrostatic forces between the cationic backbone and negatively charged groups within mucin proteins [23]; another study showed its potential to accumulate in tumour cells [22]. However, chitosan is limited by its low solubility in non-acidic environments, high viscosity in dilute aqueous acidic environments, limited drug release, and low encapsulation efficiency [19, 22, 24]. Instead, chitooligosaccharides (COS), an oligomer of chitosan that can be prepared by depolymerization of chitosan with nitrous acid or hydrochloric acid, has been explored as an alternative that retains chitosan’s desirable physical and chemical properties, while also having better solubility at relatively higher pH [24]. Control over average oligomer length of nitrous acid depolymerization has been shown to be controllable through the stoichiometric ratio between chitosan and nitrous acid [24–26]. This depolymerization also results in a reactive aldehyde group on one end of COS oligomers, which can be functionalized with a variety of groups or moieties.

The properties of synthetic polymers and natural polymers as nanomaterials compliment each other; the former provides tunability of drug release and simple nanostructure synthesis, while the latter provides customization of functional groups and targeting moieties for improved targeting and properties tailored to specific physiological environments. Recent studies have created hybrid nanostructures of synthetic and natural polymers, with synthetic polymers acting as the core structure and natural polymers acting as a coating, indirectly bound by adsorption and intermolecular forces [21, 23, 27]. Some studies have explored PLGA core, chitosan-coated NPs as potential drug nanocarriers [27, 28].

Reliance on physical adsorption for coating of natural polymers may result in decreased stability and sensitivity to changes in pH, ions, temperature, and/or solvents. In contrast, chemical conjugation via forms of linker chemistry can be more resilient, as well as less reliant on physical and electrostatic compatibility of polymer moieties. Amine-reactive crosslinker chemistry can allow for relatively straightforward and facile chemical conjugation because the reaction is regioselective and occurs under benign conditions (aqueous, room temperature, moderate pH) [29]. NHS ester groups, which can be created by modifying carboxyl groups with EDC [N-(3-Dimethylaminopropyl)-N′-ethylcarbodiimide hydrochloride] and stabilized with NHS (N-hydroxysuccinimide), are often used as an amine-specific functional group for linking or labelling [30].

### Integrins and active targeting

While chitosan has been shown to have potential for passive targeting of tumour cells, various ligands can be used in active targeting. Integrins are receptors found in the extracellular matrix (ECM), and are mainly involved in cell-cell and cell-ECM adhesion interactions [31]. Of these integrins, the α_v_β_3_ integrin is among the most important in tumour angiogenesis, and is overexpressed in several tumour types including ovarian and breast cancers [32]. The α_v_β_3_ integrin serves as a receptors to many ECM molecules that contain the Arg-Gly-Asp (RGD) peptide sequence; as such, ligands based on the RGD peptide, as well as RGD mimetics, have been shown to deliver targeted therapeutic doses of anticancer drugs like Paclitaxel and Doxorubicin to tumour cells [31, 33–35]. The presence of -COOH carboxyl groups in RGD peptide allows for conjugation to COS via NHS-amine reactive chemistry. This allows COS-coated PLGA-core NPs to be functionalized with RGD peptide for active targeting and drug delivery.

**FIGURE 1:**
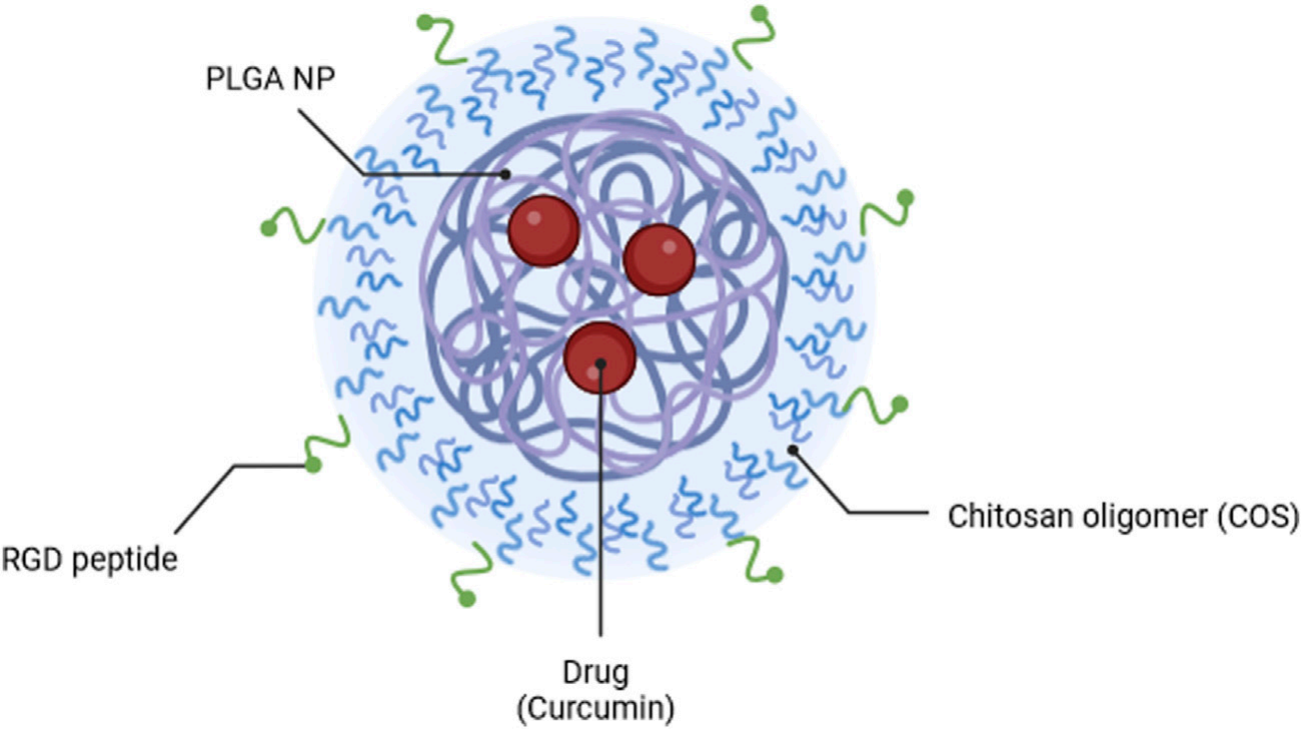
Illustration of PLGA-COS-RGD NP encapsulating curcumin drug. Created in Biorender.com.

The main objective of this study was to develop a modular nanocarrier system utilising facile assembly and conjugation methods that could used with interchangeable core and coating materials, to accelerate successful development of chemotherapy agents. NPs were assembled through the commonly used emulsion method, with PLGA as the core material, and curcumin as the encapsulated model drug. These NPs were coated with COS and RGD in tandem to promote targeting of cancer cells. Figure 1 illustrates the proposed formation of these NPs Physical properties of coated NPs were compared to bare PLGA NPs, and the formulations were compared *in vitro* via fluorescent cell uptake study and cytotoxicity assay with OVCAR-3 human ovarian tumour cells. This project hypothesizes that PLGA NPs will successfully encapsulate curcumin to protect it from degradation and provide prolonged release, and that NPs coated with COS and RGD will provide better uptake and cytotoxic effects compared to bare PLGA NPs.

## Materials and methods

### Materials

Adipic acid dihydrazide, ammonium hydroxide solution (28.0–30% NH_3_ basis), ammonium acetate, chitosan (low molecular weight; 50,000–190,000 Da, 75–85% de-acetylated), curcumin [≥94% (curcuminoid content), ≥80%], dimethyl sulfoxide (DMSO), glacial acetic acid, hydrochloric acid (37%) (HCl), MES (4-Morpholineethanesulfonic acid), methanol, methylene chloride (DCM), N-(3-Dimethylaminopropyl)-N′-ethylcarbodiimide hydrochloride (EDC), N-Hydroxysuccinimide (NHS), RGD peptide (Arg-Gly-Asp), sodium cyanoborohydride (NaBH_3_CN), sodium nitrite (NaNO_2_), trehalose dihydrate were all purchased from Sigma Aldrich. PLGA-COOH [poly (lactic-co-glycolic) acid] (50:50, and 40 K MW) was purchased from Nanosoft Polymers. Cytiva HyClone™ RPMI 1640 Media with Glutamine, Gibco™ Fetal Bovine Serum (certified, heat inactivated), Gibco™ Penicillin-Streptomycin, Phosphate-buffered saline (PBS, 1X, pH 7.4), Pierce Micro BCA Protein Assay kit, Promega CellTiter 96™ AQueous One Solution Cell Proliferation Assay (MTS) were purchased from Thermo Fisher.

### Functional chitooligosaccharide synthesis

**FIGURE 2:**
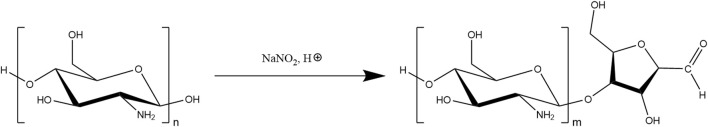
Nitrous acid depolymerization of chitosan, forming 2,5-anhydro-D-mannofuranose (amf) moiety, which contains a reactive aldehyde group.

COS was prepared from chitosan through nitrous acid depolymerization (shown in Figure 2) based on the protocols by Moussa [24, 26]; low MW chitosan was dissolved in 0.15 M HCl (2 g chitosan per 100 mL solution), then vacuum degassed to remove oxygen from the solution. 500 mM NaNO_2_ aqueous solution was prepared (with degassed MilliQ water, immediately before addition to chitosan solution), which was then added for a GlcN:NaNO_2_ molar ratio of 10:1 (to achieve COS with approximately 20 average GlcN units), before being stirred moderately overnight so as not to introduce excess oxygen to the mixture. The mixture was filtered to remove undissolved chitosan and other impurities, before precipitating COS by addition of ammonium hydroxide until reaching solution pH 9. To further increase yield, an equal volume of methanol was added to the mixture, which was then centrifuged for 10 min at 10,000 g and 4°C. The COS was washed (x3) by repeated centrifugation in 50/50 water/methanol, before a final wash step with water. The COS was then resuspended in 10 mL water and lyophilized.

**FIGURE 3:**
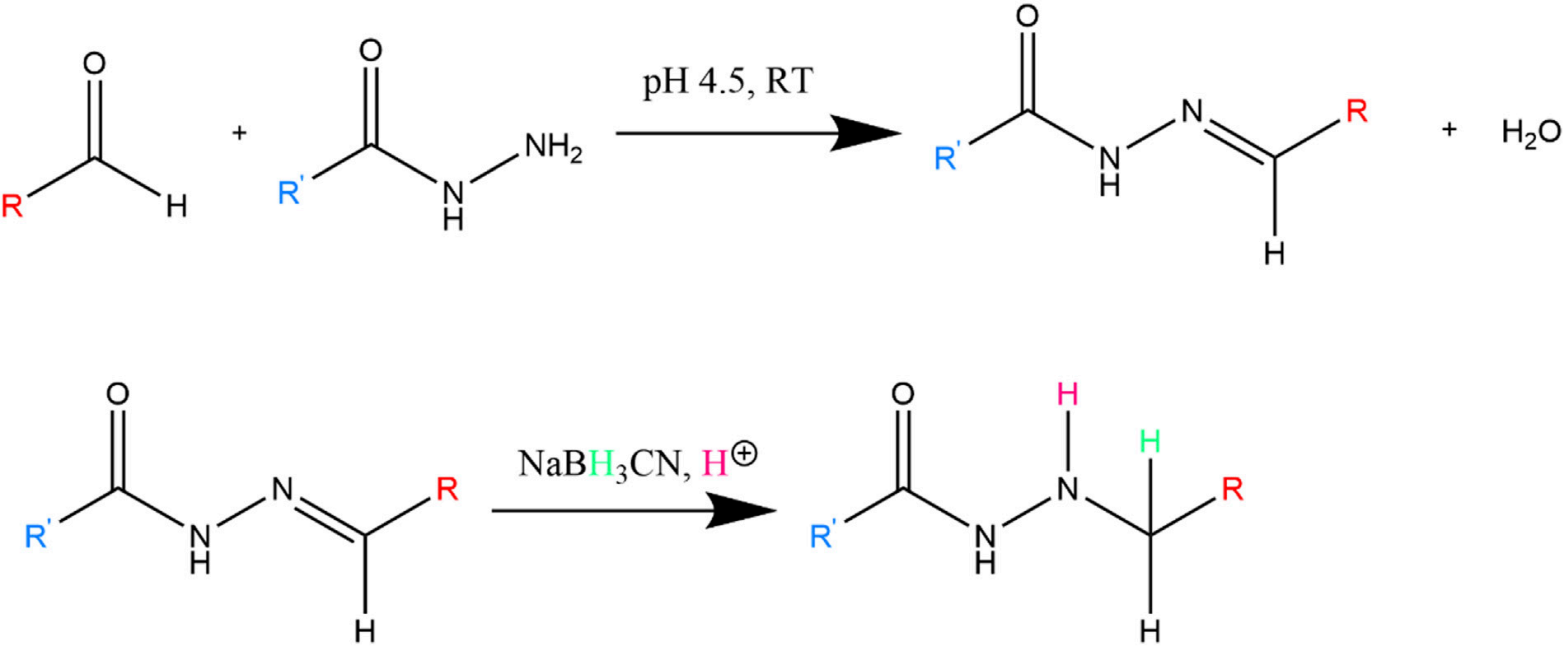
Reaction between hydrazide and aldehyde, which forms hydrazone conjugate. The hydrazone double bond is further reduced to a more stable secondary amine by NaBH_3_CN.

Hydrazide functionalized COS (COS-hydrazide) was prepared by dissolving COS in water, with additions of acetic acid. Concentrated MES was then added to a concentration of 0.15 M, before adjusting the solution to pH 4.5 with NaOH or HCl. 10 M equivalents (to COS) of adipic dihydrazide was added to solution and stirred at RT for 24 h, then 10 M equivalents (to COS) of sodium cyanoborohydride was added to solution and stirred at RT for 24 h. As depicted in Figure 3, the COS aldehyde functional group reacts with the hydrazide under acidic conditions to form a hydrazone conjugate, which is further reduced to a secondary amine bond by sodium cyanoborohydride. The COS-hydrazide solution was filtered, precipitated, washed and lyophilized following the same protocol as COS. COS and COS-hydrazide were analysed with ^1^H NMR spectroscopy to confirm average size and presence of aldehyde or hydrazide groups, respectively. ^1^H NMR spectroscopy was performed on Bruker 500 MHz spectrometer at 300 K. Samples were diluted in deuterated solvent (D2O) to 10 mg/mL; 5 μL HCl (12 M) was added to 1000 μL COS samples to facilitate dissolution in D2O. NMR spectra was analysed using Bruker TopSpin NMR data analysis software.

### Synthesis of PLGA-COS NPs

Void (without encapsulated drug) PLGA NPs were prepared by emulsion method, based loosely on the works of Rafiei and McCall [17, 36]. The initial emulsion mixture consisted of 1:4 v/v organic to aqueous parts. The organic portion was PLGA (acid-terminated) dissolved in DCM to a concentration of 50 mg/mL, while the aqueous portion was 3% w/v PVA. The mixture was briefly vortexed, then emulsified under ice bath via tip sonication. Tip sonication of NP samples were performed with QSonica Q125 Sonicator. Samples were sonicated at 60% amplitude, at 15 s ON, 5 s OFF pulse, for 90 s. The tip was moved around in sample solution during initial sonication pulse to ensure all the organic portion was fully emulsified. The emulsified mixture was then diluted with 1% PVA solution to 300% of its original volume and stirred for 3 h to evaporate the organic solvent. The NP mixture was centrifuged (15,000 g, 10 min, 4°C) and washed with water (x3) to remove excess PVA, before being resuspended in 5 mL DI water (and 5% w/v trehalose as cryoprotectant if necessary) and frozen for lyophilization. A small aliquot was reserved for measuring NP size and zeta potential (ZP). Particle size/distribution and zeta potential of NPs were respectively measured by dynamic light scattering (DLS) and electrophoretic light scattering (ELS) using Malvern Zetasizer Ultra-Red.

**FIGURE 4:**
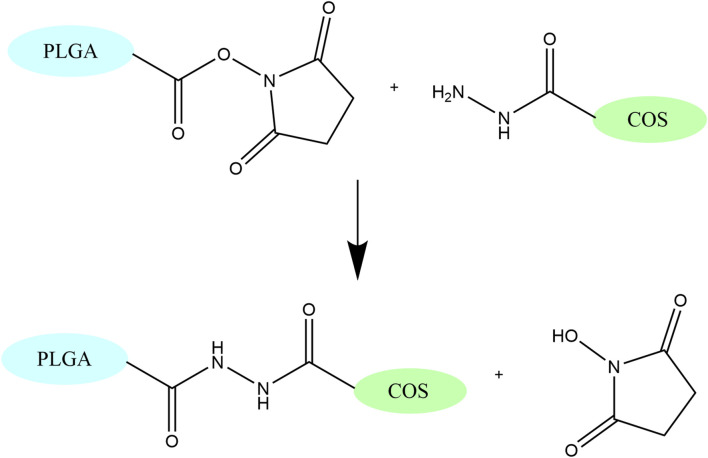
Secondary amide formation between NHS and hydrazide group, which links COS to PLGA. This reaction occurs under acidic conditions (pH 4-5).

PLGA-COS NPs were formed by chemically conjugating COS-hydrazide to the surface of PLGA NPs using amine-reactive NHS chemistry (see Figure 4). COS-hydrazide was dissolved in a small amount of dilute o-phosphoric acid, before being added to MES buffer (100 mM, pH 5) to a COS concentration of 5 mg/mL. PLGA NPs were added resuspended in this solution (2:1 PLGA:COS w/w), along with NHS and EDC (approx. 20%, 30% w/w PLGA NPs, respectively). The mixture was stirred vigorously overnight before washing excess NHS, EDC, and COS through centrifugation. A small aliquot was reserved for measuring NP size and ZP, while the rest was prepared for lyophilization as previously described with PLGA NPs.

### Synthesis of PLGA-COS-RGD NPs

RGD peptide was chemically conjugated to COS amine groups through amine-reactive NHS chemistry. 1 mg RGD peptide was added to 10 mL MES buffer (100 mM, pH 6.5) along with 0.5 mg NHS, and 2 mg EDC. PLGA-COS NPs were added and resuspended in the buffer solution through vortexing (and mild sonication if necessary). The NP mixture was stirred overnight, before being centrifuged to separate NP from free RGD. BCA assay was performed on samples of supernatant to determine binding efficiency of RGD to the NPs. NHS concentration across all samples and standards were kept constant for BCA assay analysis, due to the affect and interference of NHS (by reducing the Cu compound in BCA reagent). BCA and other colourimetric assays were recorded and analysed using Varioskan LUX 3020 Multimode Microplate Reader.

### PLGA-CUR NPs

#### Synthesis

Curcumin-encapsulated PLGA NPs (PLGA-CUR NPs) were prepared by emulsion method, similar to void PLGA NPs (*Synthesis of PLGA-COS NPs*). Curcumin was dissolved in DCM alongside PLGA (1:10 curcumin: PLGA, w/w), then added to the aqueous solution containing PVA. After emulsion and stirring to evaporate DCM solvent, the NP mixture was filtered through Whatman Grade 1 filter paper to remove precipitated and aggregated curcumin, before centrifugation and washing steps.

#### UPLC analysis

Analysis of curcumin content was performed using UPLC, using Waters Acquity UPLC H-Class Plus system. The column used was Waters ACQUITY UPLC HSS C18 Column (100 Å, 1.8 μm, 2.1 mm × 100 mm; pore size, diameter, dimensions). Mobile phases were as follows: phase A – acetonitrile (ACN) (100%); phase B – 10% acetonitrile, 90% 10 mM o-phosphoric acid aqueous buffer. For sample analysis, flow parameters of each run were kept at constant flow rate of 0.4 mL/min, with gradient mobile phase composition. Percentage composition is given as XX% (by volume) mobile phase A; the remaining mobile phase is composed of mobile phase B. From 0 to 0.5 min, 44.4% A. From 0.5 to 1.0 min, gradient increases from 44.4% to 77.7% A. This gradient is held until 3.5 min. From 3.5 to 4 min, gradient is decreased back to 44.4% A, and held for the rest of the run. PDA UV-Vis absorbance detector was set at 430 nm.

#### Encapsulation efficiency

Due to the hydrophobic nature of curcumin, indirect measurement of encapsulation efficiency (EE) and drug-loading efficiency (DL) through the supernatant post-synthesis was not feasible. Instead, a known mass of lyophilized curcumin-loaded NPs were dissolved in organic solvent. These samples were analysed via UV-Vis absorbance plate reader and compared against a standard curve of curcumin (100–10,000 ng/mL) dissolved in ACN. The calculated concentration was compared to the initial amount of curcumin and theoretical maximum encapsulation concentration (10% NP mass, based on the synthesis parameters) to determine EE% and DL%, respectively.

$$\text{EE}\%=\frac{\text{Measured}\;\text{encapsulated}\;\text{curcumin}\;\text{conc}.}{\text{Maximum}\;\text{encapsulated}\;\text{curcumin}\;\text{conc}.}*100\%$$

$$DL\%=\frac{\text{Measured}\;\text{encapsulated}\;\text{curcumin}\;\text{mass}}{\text{Total}\;\text{NP}\;\text{mass}}*100\%$$

#### Degradation and release study

Curcumin-encapsulated NPs were resuspended in 50 mL PBS buffer (pH 7.2) to 2500 ng/mL (or 500 ng/mL) curcumin, using DL% of the NPs to find the required mass of NPs to achieve desired concentration. This solution was stirred, and 1 mL aliquots were taken at various time points for up to 72 h. These aliquots were treated based on the experiment: either curcumin within only the supernatant, only the NP pellet, or the entire solution was measured. Degradation of curcumin in NPs vs. free curcumin was measured within the entire solution; this simply required dilution with ACN followed by centrifugation (20,000 g for 5 min) to remove any insoluble precipitate. Samples were then transferred to UPLC vials for analysis. Supernatant or NP pellet samples were prepared by separating the two via centrifugation (20,000 g for 5 min), to separate free curcumin from NP pellet. The supernatant was then mixed with ACN to match UPLC mobile phase composition and transferred to UPLC vials. If NP pellet content was to be measured, ACN was added to fully dissolve PLGA NPs, then diluted with buffer to mobile phase composition and transferred to UPLC vials. Curcumin was quantitated against a standard calibration curve from 50 to 4000 ng/mL.

### Cell uptake study

The cell uptake study was designed to assess the effectiveness of the COS-RGD-coated NPs at targeting tumour cells versus bare PLGA NPs. RGD peptides have previously been shown to effectively target OVCAR-3 human ovarian carcinoma cells; thus the cell line was used in this study [37]. To better visually compare treatments, Coumarin-6, a hydrophobic fluorescent dye, was used as a replacement encapsulant to curcumin. Coumarin-6 achieves peak excitation around 457 nm.

Briefly, OVCAR-3 cells were cultured and then seeded in a 96-well plate, letting cells incubate for 24 h at 37°C and 5% CO_2_. Coumarin-6 in PLGA NPs, PLGA-COS NPs, PLGA-COS-RGD NPs, or free Coumarin-6 (1000 ng/mL) was suspended in cell medium and added to the wells, incubating for another 24 h under the same conditions. The plates were washed with PBS solution, then cells were fixated with formaldehyde solution (4%), and finally stained with DAPI solution (300 nM) for 5 min. The plates were once again washed with PBS and then imaged with fluorescent microscopy. The DAPI stain and Coumarin-6 each showed up under separate channels. ImageJ was used to quantify average fluorescent intensity of Coumarin-6 within cells to determine relative uptake.

### Cell toxicity study

As a further demonstration of the delivery effectiveness of PLGA NPs, OVCAR-3 cells were treated with NP-encapsulated and free curcumin for up to 24 h. The different treatments were compared with varying curcumin concentrations (between 2.5 and 40 μg/mL) and treatment times (between 2 and 24 h). To isolate the effects of the delivered curcumin and the NPs themselves, OVCAR-3 cells were also treated for 24 h with void NPs, at the highest and lowest void NP concentrations equivalent to the loaded NP treatments. This void treatment would help determine if the NPs were contributing to cell toxicity directly, in addition to their delivery of curcumin drug.

OVCAR-3 cells were seeded in a 96-well plate at 5000 cells/well and incubated for 24 h at 37°C and 5% CO_2_. Treatment medium was produced by resuspending NPs in cell culture medium; free curcumin treatment medium to assist in dispersion of curcumin, which was insoluble in the aqueous medium. After incubation of cells, old medium was replaced with treatment medium, and the cells were incubated under the same conditions as prior (for 24 h in all non-time-dependent studies). After treatment incubation, cells were washed several times with PBS to remove residual curcumin; MTS assay was then performed with Promega CellTiter 96™ AQueous One Solution Cell Proliferation Assay, following the recommended protocol. Colourimetric results from the MTS assay were quantified with Varioskan microplate reader.

### Statistical analysis

All experiments were conducted in triplicate. Data was statistically analysed by unpaired t-test and one-way ANOVA where applicable. In cases where ANOVA found statistical significance, Tukey’s test was used to determine which groups contained statistical differences. The value of α = 0.05 was used to determine whether results were statistically significant.

## Results

### Chitosan oligomer characteristics

**FIGURE 5:**
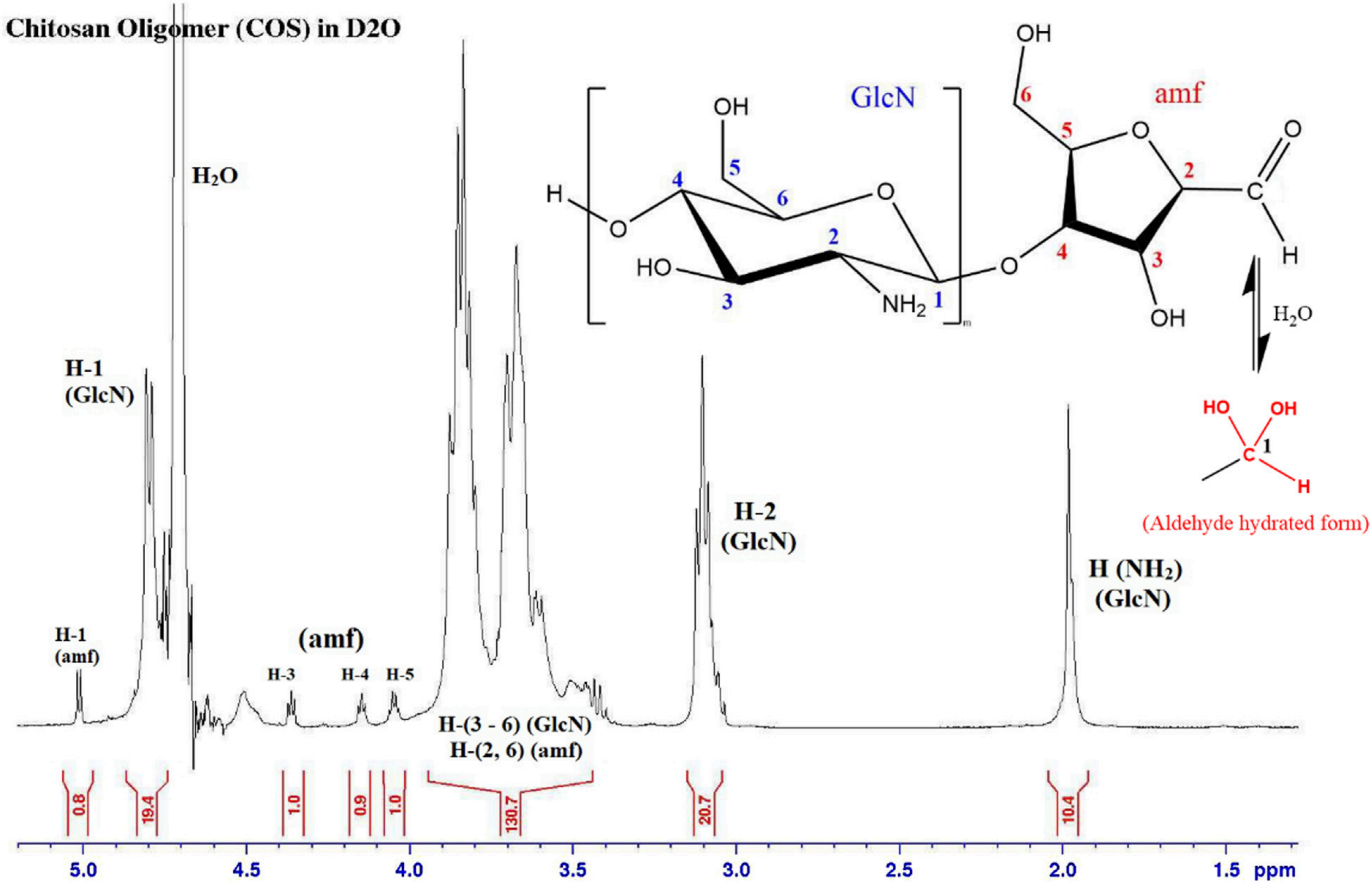
^1^H NMR spectrum of COS sample, with GlcN to amf ratio of 20. The aldehyde proton (H-1 amf) shows up in its hydrated form.

Depolymerization was successfully confirmed with ^1^H NMR spectroscopy. The presence of the amf end unit is confirmed by the small peak at 5.01 ppm that corresponds to the aldehyde proton (specifically the hydrated form of the aldehyde), as well as peaks at 4.05, 4.15, and 4.36 ppm ^1^H spectra are corroborated by NMR data of COS from the Moussa study [24, 26]. By comparing the H-2 GlcN to H-1, 3, 4 or 5 amf proton signals in Figure 5, we can see that the average length of each COS molecule is around 20 GlcN units to every amf end unit. Control of the average length of COS was shown to be relatively replicable; average of triplicate samples of COS using a 10:1 GlcN/NaNO_2_ molar ratio showed an average observed length of 21.8 ± 1.8 units. Stoichiometric control was further confirmed by increasing the amount of NaNO_2_ and observing a change in average oligomer length; average of triplicate samples of COS using a 10:1 GlcN/NaNO_2_ molar ratio showed an average observed length of 10.6 ± 2.4 units.

**FIGURE 6:**
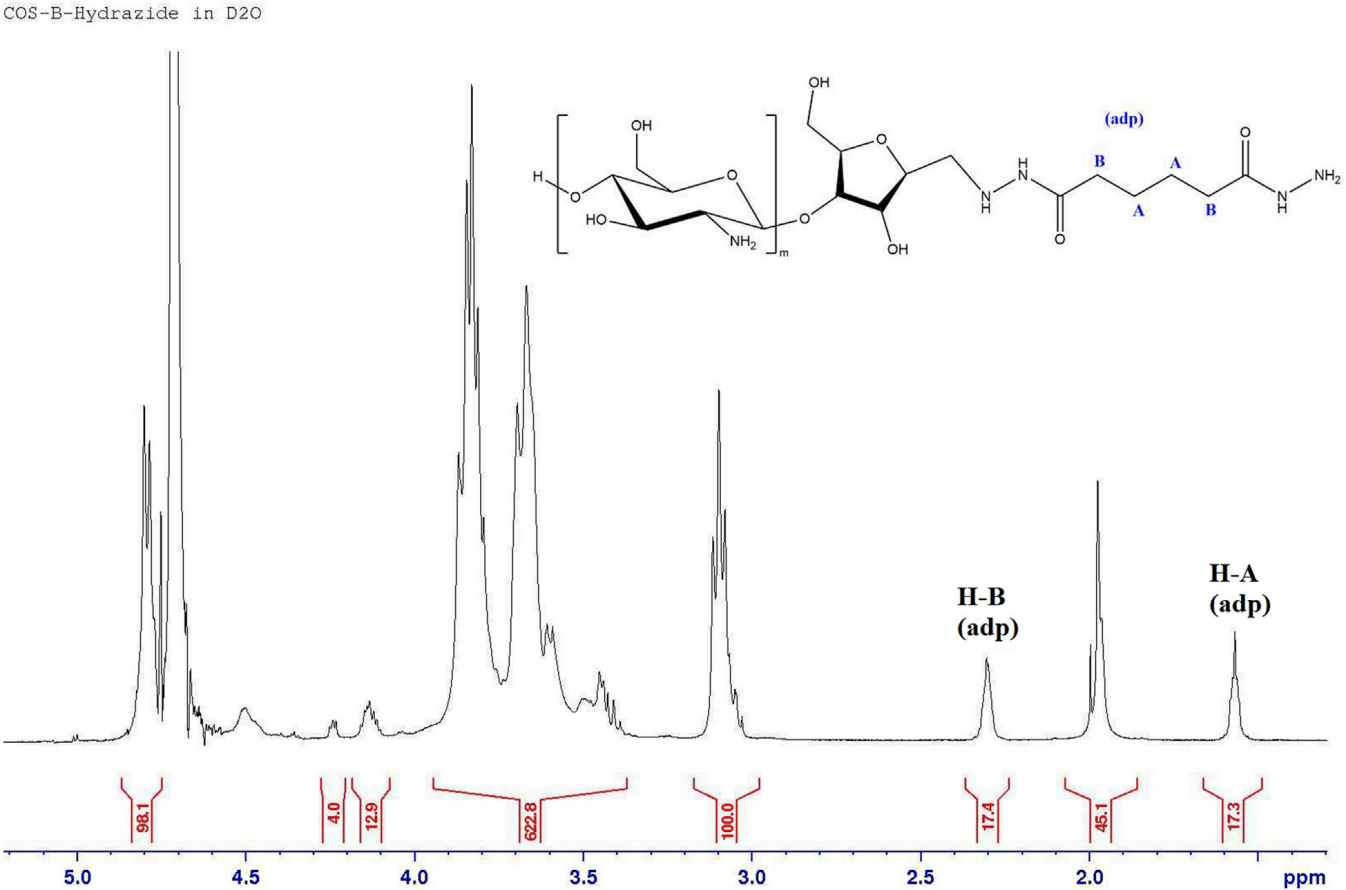
^1^H NMR spectrum of COS-hydrazide sample.

The successful attachment of adipic hydrazide to COS through the amf aldehyde group is confirmed by the absence of the hydrated aldehyde peak (5.01 ppm) as the reaction site, as well as the presence of peaks at 1.57 and 2.31 ppm that correspond to the aliphatic carbon chains between the hydrazide groups, as seen in Figure 6.

### NP characteristics

PLGA NPs were successfully synthesized by emulsion method; their characteristics were replicated by maintaining consistent parameters including polymer concentration, organic to aqueous ratio, PVA content, and sonication intensity. Similarly prepared PLGA-COS NPs were also prepared, and both were characterized by size, PDI and ZP (zeta potential) via Zetasizer (results shown in Table 1). Both PLGA and PLGA-COS NPs have a similar size (less than 300 nm average), and a relatively narrow PDI (less than 0.05). The COS coating did not appear to affect the size or PDI of the NPs significantly. The negative ZP of PLGA NPs is mainly due to the carboxyl end groups of PLGA molecules at the surface of the NPs. The positive ZP of PLGA-COS NPs suggests the successful coating of COS to the PLGA NP surface, due to the presence of positively charged amine groups in COS. However, PLGA COS NPs could not be directly characterized via ^1^H NMR, as the NPs encountered solubility issues -- likely due to the fact that the PLGA portion is highly soluble in organic solvents while the COS portion is highly insoluble in organic solvents, and requires relatively acidic conditions to facilitate solubility even in aqueous media.

**TABLE 1:** Zetasizer results (n = 3) of PLGA, PLGA-COS NPs.

	Z-avg size (d, nm)	PDI	Zeta potential (mV)
PLGA NPs	221.7 ± 19.7	0.085 ± 0.054	−15.2 ± 0.7
PLGA COS NPs	229.2 ± 50.2	0.119 ± 0.056	+26.1 ± 1.2
PLGA NPs (w/curcumin)*	281.2 ± 23.4	0.238 ± 0.060	N/A
PLGA COS NPs (w/curcumin)*	278.8 ± 102.0	0.195 ± 0.080	N/A

*NP results containing encapsulated curcumin. Fluorescence of curcumin negatively affects results.

When comparing PLGA NPs with and without encapsulated curcumin (via t-test), there was statistically significant difference in NP size and PDI. Comparisons between samples of PLGA COS NPs with and without encapsulated curcumin did not conclude a statistically significant difference. It is however, important to note that curcumin exhibits some fluorescent properties, which caused interference with both the DLS and ELS techniques employed by the Zetasizer to obtain size and ZP data. Fluorescence filters were able to mitigate interference when determining size, but attempts to determine ZP were highly inconsistent. It is highly unlikely that surface charge is affected by encapsulation of drug.

NP and COS coating stability was further investigated by Zetasizer measurements of size, PDI and ZP over a 48 h period, where NP samples (unloaded) were left in aqueous buffer (PBS 1X, pH 7.2, RT) and measured in the Zetasizer at various time points (shown in Table 2). Statistical analysis (ANOVA, α = 0.05) did not show statistically significant changes to either size or ZP in either group over the full 48 h, suggesting that the NPs themselves are relatively stable over the short term, and that the positively charged COS coating remains adhered to the surface, even when dispersed in an aqueous medium.

**TABLE 2:** Zetasizer results (n = 3) of PLGA, PLGA-COS NPs over a 48 h period to assess stability.

	Time (h)	Z-avg size (d, nm)	PDI	ZP (mV)
PLGA NP	0	224.5 ± 25.3	0.025 ± 0.015	−14.4 ± 0.8
	12	246.1 ± 21.4	0.038 ± 0.011	−21.0 ± 0.8
	24	242.9 ± 22.0	0.051 ± 0.014	−17.5 ± 3.0
	48	247.2 ± 26.1	0.035 ± 0.037	−22.1 ± 6.2
PLGA COS NP	0	258.3 ± 7.5	0.102 ± 0.019	26.3 ± 0.4
	12	275.5 ± 31.2	0.124 ± 0.034	24.7 ± 0.6
	24	279.3 ± 58.3	0.034 ± 0.023	24.6 ± 0.3
	48	275.4 ± 27.1	0.137 ± 0.032	25.3 ± 4.0

**FIGURE 7:**
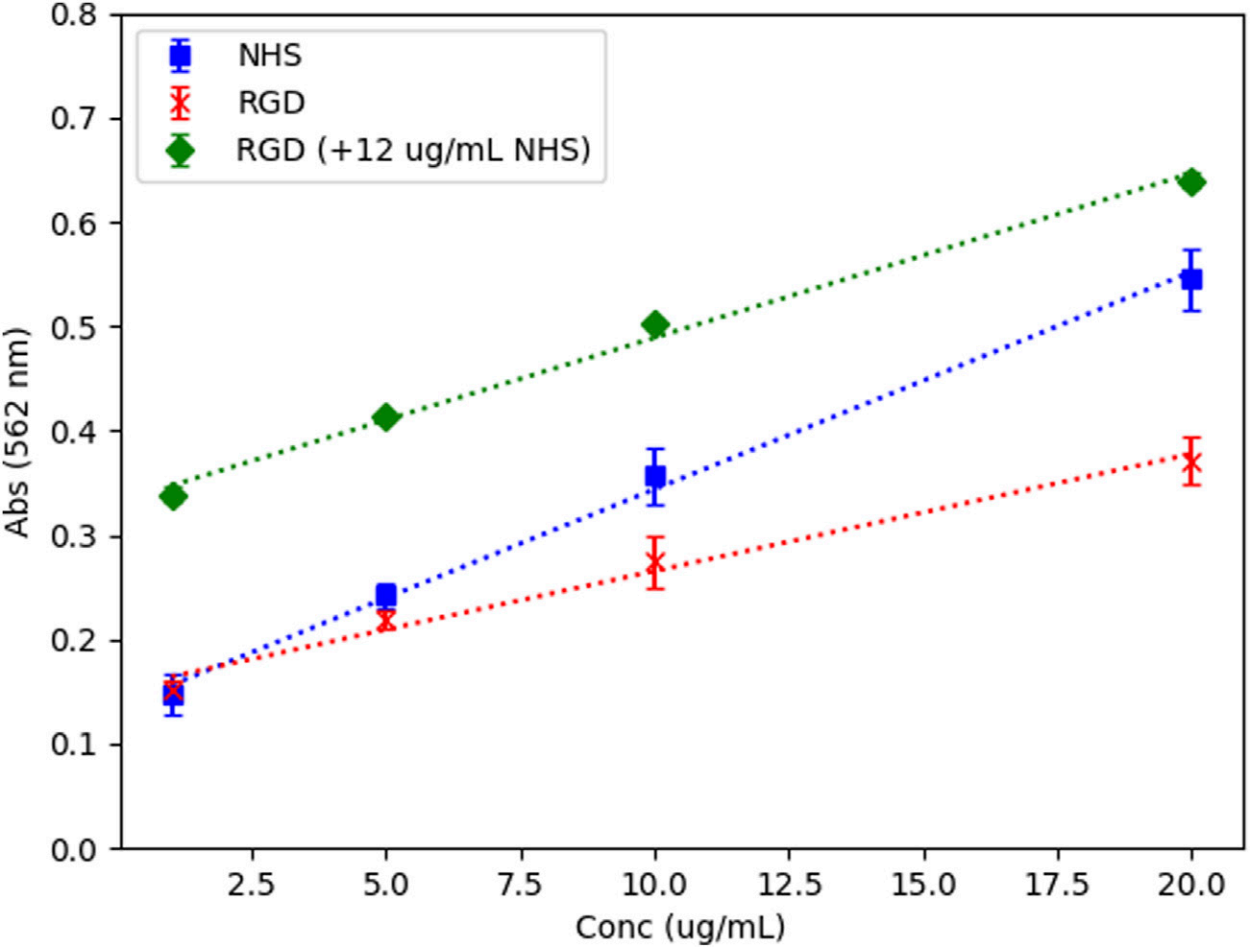
BCA assay standard curves – NHS, RGD, and RGD with controlled NHS conc. All standards prepared in 100 mM MES buffer, pH 5, N = 3. R^2^ > 0.99 for all standard curves.

RGD coating onto PLGA-COS NPs were confirmed by BCA assay of both the supernatant solution, and the NPs directly. The standard curves (Figure 7), which compared the curves of RGD peptide, NHS, and RGD peptide with a controlled concentration of NHS, confirm that NHS interference with BCA assay is consistent and predictable, and that maintaining the same concentration across samples/standards minimizes potential variance due to its presence. The supernatant samples, which contained NHS from the binding reaction, were analysed against the RGD standards containing 12 μg/mL NHS, and diluted appropriately to match this concentration. The NP samples, which were centrifuged and washed of residual reaction reagents, were analysed against RGD standards without NHS. The assays showed a 12.9% ± 0.4% binding efficiency.

### Curcumin-loading results

#### Drug loading and encapsulation efficiency

Samples of curcumin-loaded PLGA NPs were dissolved in 3 different organic solvents (ACN, ethyl acetate, chloroform) to ensure full extraction of curcumin from NPs. Each sample was dissolved to an initial concentration of 1 mg/mL (NP mass) and performed in triplicate. At 100% theoretical EE, the NPs would contain 10 μg/mL curcumin. The results are shown in Table 3. Regardless of solvent as dissolution medium, the results indicate just under 6 μg/mL curcumin, which equates to 6% drug loading (DL) or 60% encapsulation efficiency (EE). These encapsulation efficiency results were below expected values based on similar studies, but within acceptable range for subsequent testing [38, 39]. Degradation of curcumin during synthesis, processing, or storage may account for some of this discrepancy.

**TABLE 3:** Curcumin drug loading of PLGA NPs through direct dissolution of NP (n = 3).

Sample (solvent)	Avg conc (μg/mL)	SD (μg/mL, n = 3)	DL% (%)	EE% (%)
Acetonitrile	5.58	0.13	5.58	56
Ethyl Acetate	5.95	0.04	5.95	59
Chloroform	5.93	0.15	5.93	59

#### Degradation and release study

**FIGURE 8:**
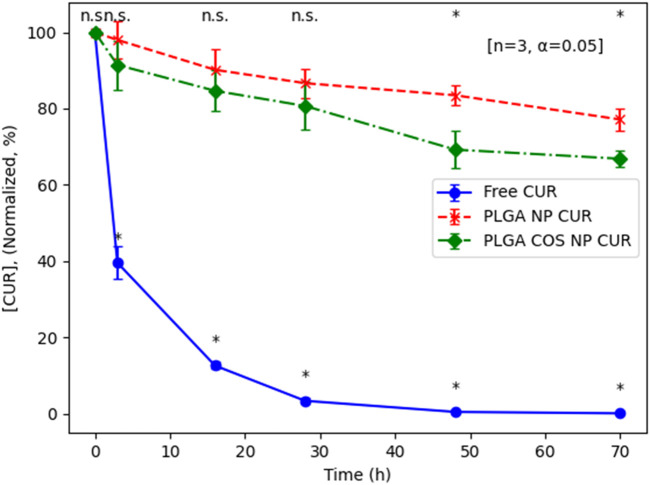
UPLC results of degradation of encapsulated curcumin compared to free curcumin in PBS solution (pH 7.2, RT). Analysis via t-test (n = 3, α = 0.05; (*): p < 0.05) shows some significant between curcumin (CUR)-loaded PLGA NPs and PLGA COS NP at 48 h and 72 h (marked at top). Free curcumin is shown to degrade significantly compared to the NP samples even after only 3 h (marked at bottom).

To determine the difference in degradation of curcumin encapsulated in NPs and curcumin free in aqueous solution, triplicate samples of free curcumin, PLGA NP encapsulated curcumin, and PLGA-COS NP encapsulated curcumin were prepared by suspending lyophilized loaded NPs in PBS 1X (pH 7.2) to a curcumin concentration of 2500 ng/mL (free curcumin samples were prepared by first dissolving curcumin in ACN, then adding to PBS and placing under vacuum to assist in evaporation of organic solvent). These samples were allowed to spin at RT for 70 h; aliquots were taken at various time points and analysed via UPLC. These aliquots were mixed with organic solvent (ACN) to break the NPs to release and dissolve the curcumin. After 70 h, it is clear encapsulated curcumin is resistant to aqueous degradation compared to the free curcumin control group, as seen in Figure 8. By 70 h, PLGA and PLGA-COS NP-encapsulated curcumin degraded to 77% and 66% of its original concentration, respectively; meanwhile, free curcumin degraded to the point where it was far below the lowest concentration standard, nearing <1% of its original concentration. PLGA-COS NP did slightly underperform relative to PLGA NP samples, though this could be due to the relatively prolonged processing to add COS-coating affecting encapsulation characteristics. Based on t-tests comparing sample groups at the same time points, the difference between PLGA NP and PLGA-COS NP samples is only statistically significant around 48 h.

### Cell uptake study

**FIGURE 9:**
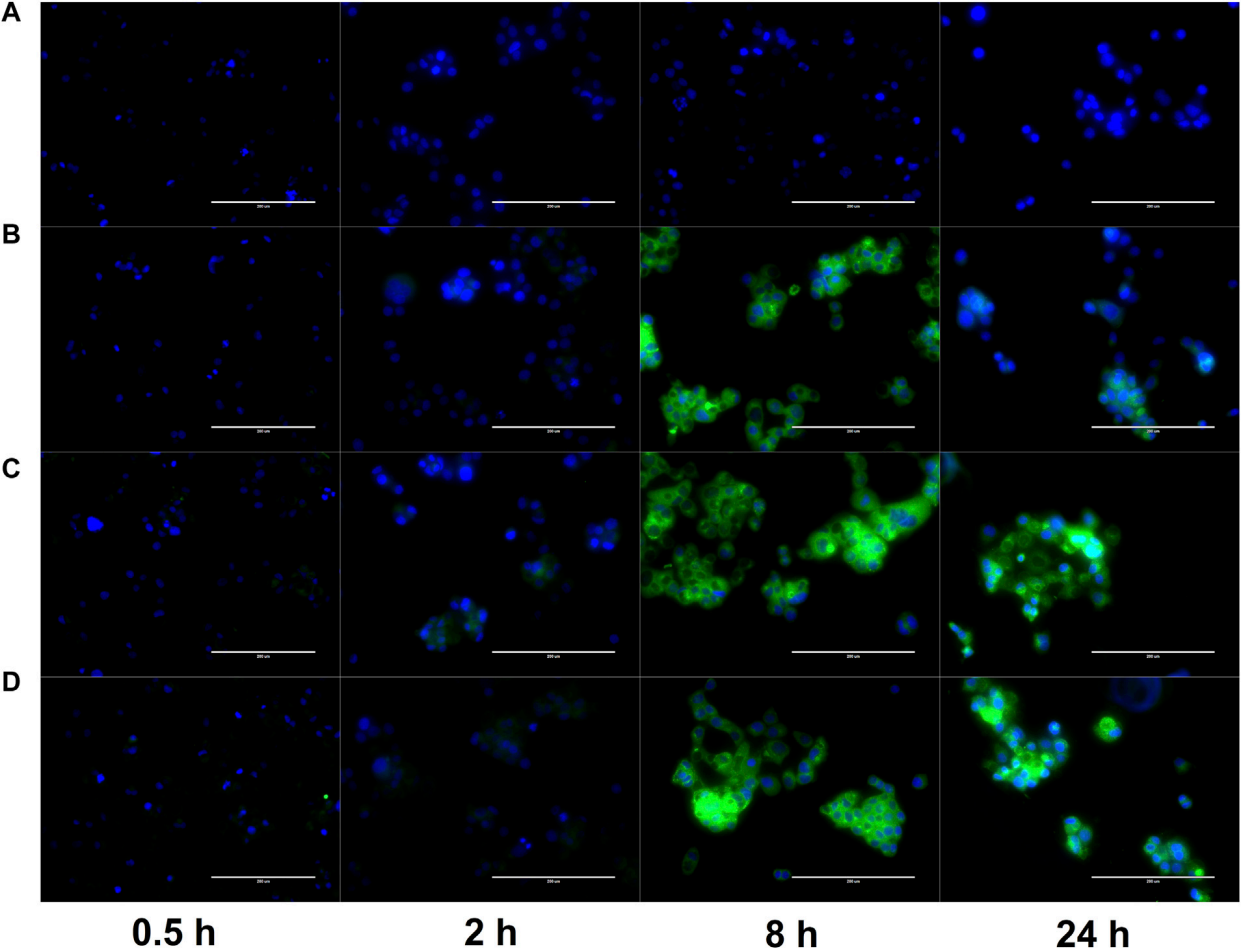
FLR microscopy of uptake of Coumarin-6 fluorescent dye (green) by OVCAR-3 cells. Cells incubated in cell medium with Coumarin-6 encapsulated PLGA NPs for up to 24 h. Cell nuclei stained with DAPI (blue) to contrast. Groups **(A–D)**: {A-free Coumarin-6 (control); B-PLGA NP; C-PLGA-COS NP; D-PLGA-COS-RGD NP}. White bar in each image represents 200 µm.

Cell uptake of PLGA NP-encapsulated hydrophobic fluorescent dye Coumarin-6 was monitored at various time points up to 24 h. This uptake was compared to a control group with free Coumarin-6. As shown in Figure 9, free Coumarin-6 (Group A) was unable to enter the cell even after 24 h of incubation. Meanwhile, Groups B-D (PLGA NP, PLGA-COS NP, PLGA-COS-RGD NP samples respectively) all showed faint fluorescent signal, and therefore successful uptake, even after 0.5 h of incubation.

**FIGURE 10:**
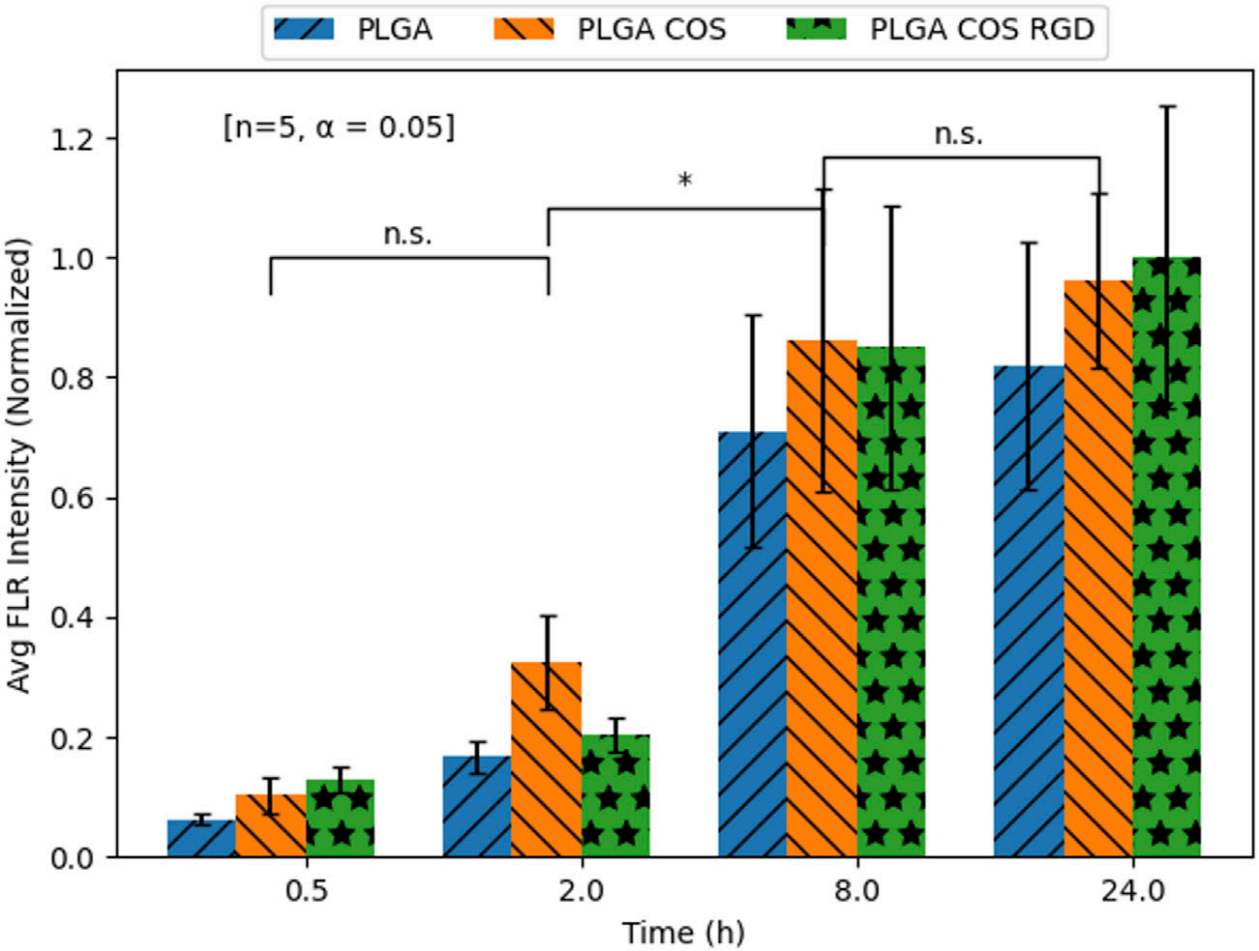
Mean Coumarin-6 fluorescent intensity values of OVCAR-3 cells treated with Coumarin-6 encapsulated NPs. Values extracted from FLR microscopy images using ImageJ analysis software. Analysis [t-test; n = 5, α = 0.05; (*): p < 0.05] did not find significant differences between the three NP treatment groups at any time point; it also only found differences within groups between 2 and 8 h.

The fluorescent images of the OVCAR-3 cells were further analysed with ImageJ software; average fluorescent intensity of an image was determined by sampling intensity at 5 different cells in each image. Results are displayed in Figure 10. Statistical analysis (t-test) determined fluorescent intensity was not significantly different from 8 h to 24 h in Groups B, C or D, suggesting near maximum uptake had been reached by 8 h. Similar analysis between Groups B, C, D showed little statistically significant difference between the overall uptake after 24 h. This seems to suggest that the coated NPs do not provide significant improved delivery over bare PLGA NPs.

### Cell toxicity study

**FIGURE 11:**
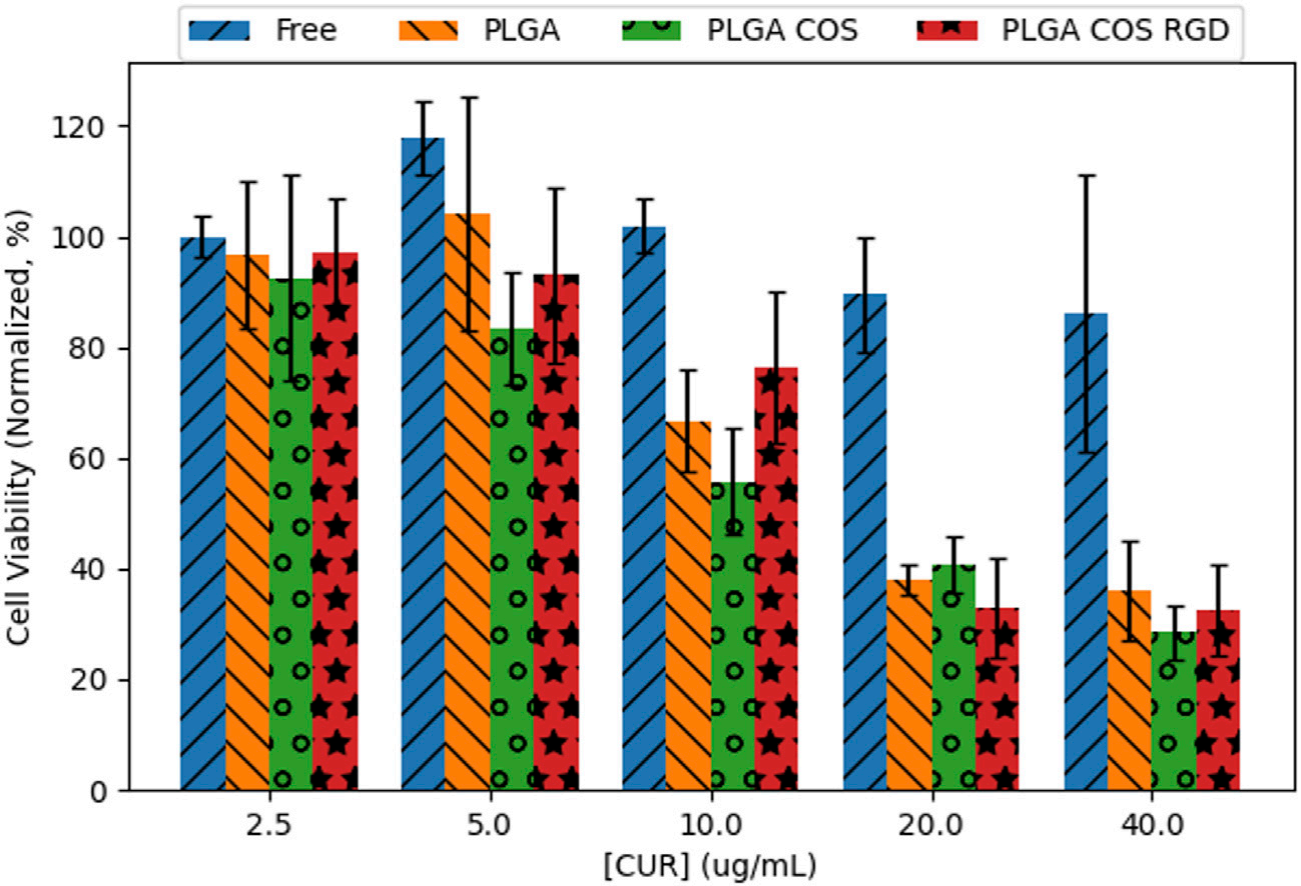
Cell viability of 24 h treatment of OVCAR-3 cells at varying [curcumin] (2.5–40 μg/mL) with MTS assay (n = 3). Statistical analysis (ANOVA, followed by Tukey’s test) did not find significant differences between the different curcumin-loaded NP groups; however, they did exhibit statistical toxicity when compared to unencapsulated free curcumin.

**FIGURE 12:**
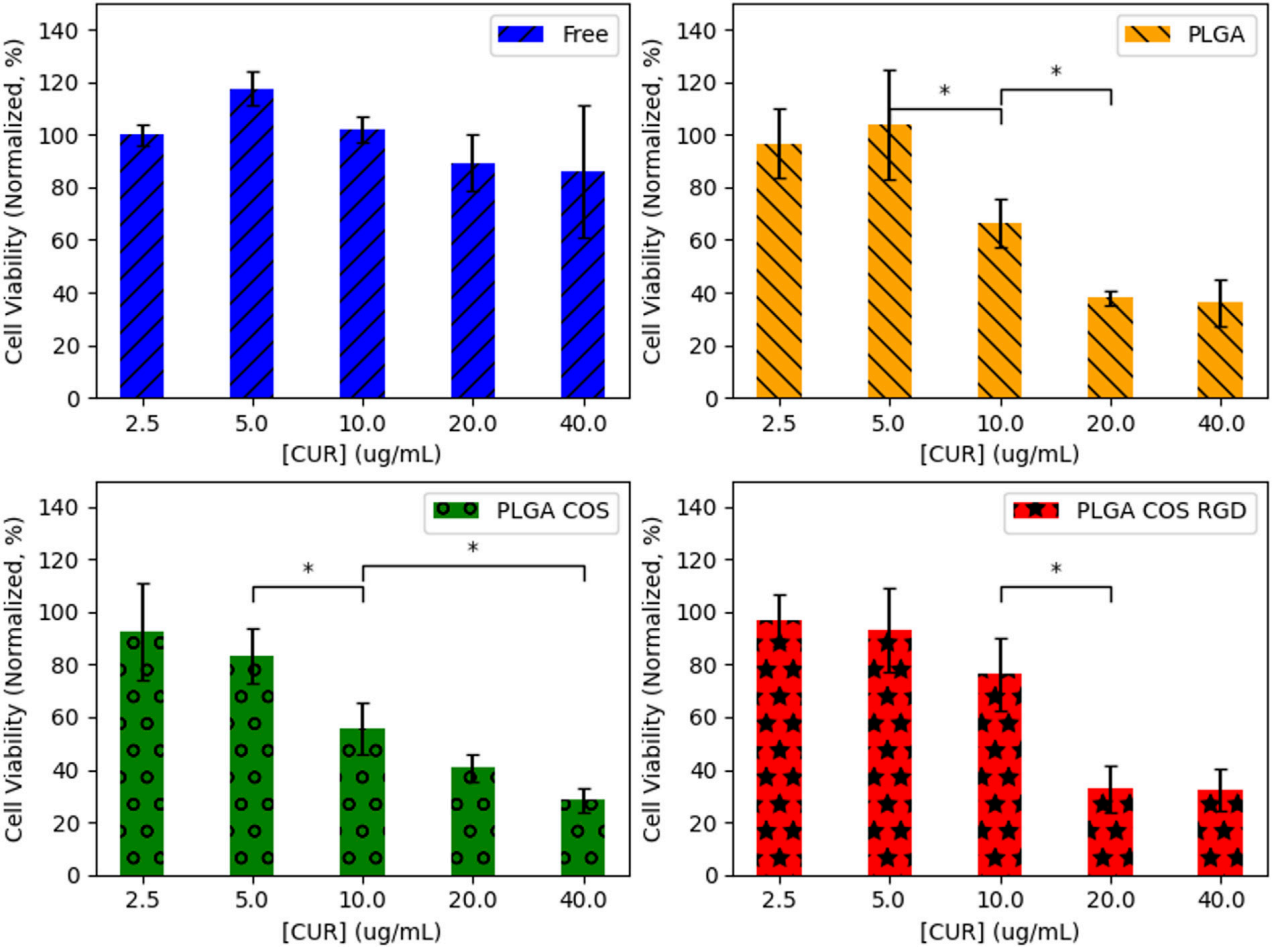
Statistical analysis (ANOVA followed by Tukey’s test) of each treatment group of OVCAR-3 cells with varying [curcumin] (t = 24 h). 95% CI (α = 0.05), n = 3. (*) denotes comparison between two concentrations where {p < 0.05}. Any adjacent concentrations without explicit comparison are assumed to be “n.s.” {p > 0.05}.

OVCAR-3 cells were treated with curcumin-loaded NPs at varying concentrations from 2.5 to 40 μg/mL (shown in Figure 11). The 3 treatment groups of PLGA NP, PLGA-COS NP, PLGA-COS-RGD NP were compared to each other and a positive control group of free, unencapsulated curcumin. Statistical analysis (ANOVA) of MTS assay indicate that change in cytotoxicity with free (unencapsulated) curcumin is not statistically significant between low and high concentrations of curcumin. Comparison of NP-encapsulated curcumin treated samples via ANOVA and Tukey’s test indicate significant difference in cytotoxicity with increasing curcumin concentration; in the 3 NP treatments, more than 5 μg/mL curcumin is required for a statistical cytotoxic effect, while cytotoxicity does not meaningfully increase beyond 20 μg/mL curcumin. IC50 (half-max inhibitory concentration) for all NP treatment groups appears to fall between 10 and 20 μg/mL; ANOVA between the three NP treatment groups did not show significant difference in their cytotoxicity. Specific statistical analysis of varying concentrations for each treatment group is shown in Figure 12.

**FIGURE 13:**
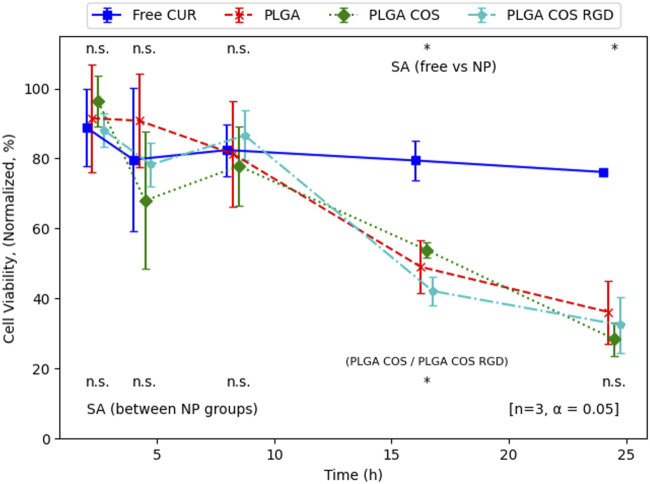
Normalized cell viability of time-dependent treatment of OVCAR-3 cells with curcumin-loaded NPs (40 μg/mL curcumin) over 24 h. Analysis (t-test, 95% CI (α = 0.05), n = 3) indicates that there is no significant difference between NP groups (except at 16 h, between PLGA COS and PLGA COS RGD samples), but there is a clear effect of the NP samples on cell viability compared to the minimal effect by free curcumin, occurring between 8 and 16 h.

OVCAR-3 cells were then treated at various with curcumin-loaded NPs for up to 24 h, at a concentration of 40 μg/mL based on the results from Figure 11. As shown in Figure 13, significant cytotoxicity began to occur after 8 h. At this concentration, cell viability appeared to reach 50% around the 16 h mark. Again, free curcumin does not appear to have statistically significant effect on the cells without the assistance of the NP delivery.

**FIGURE 14:**
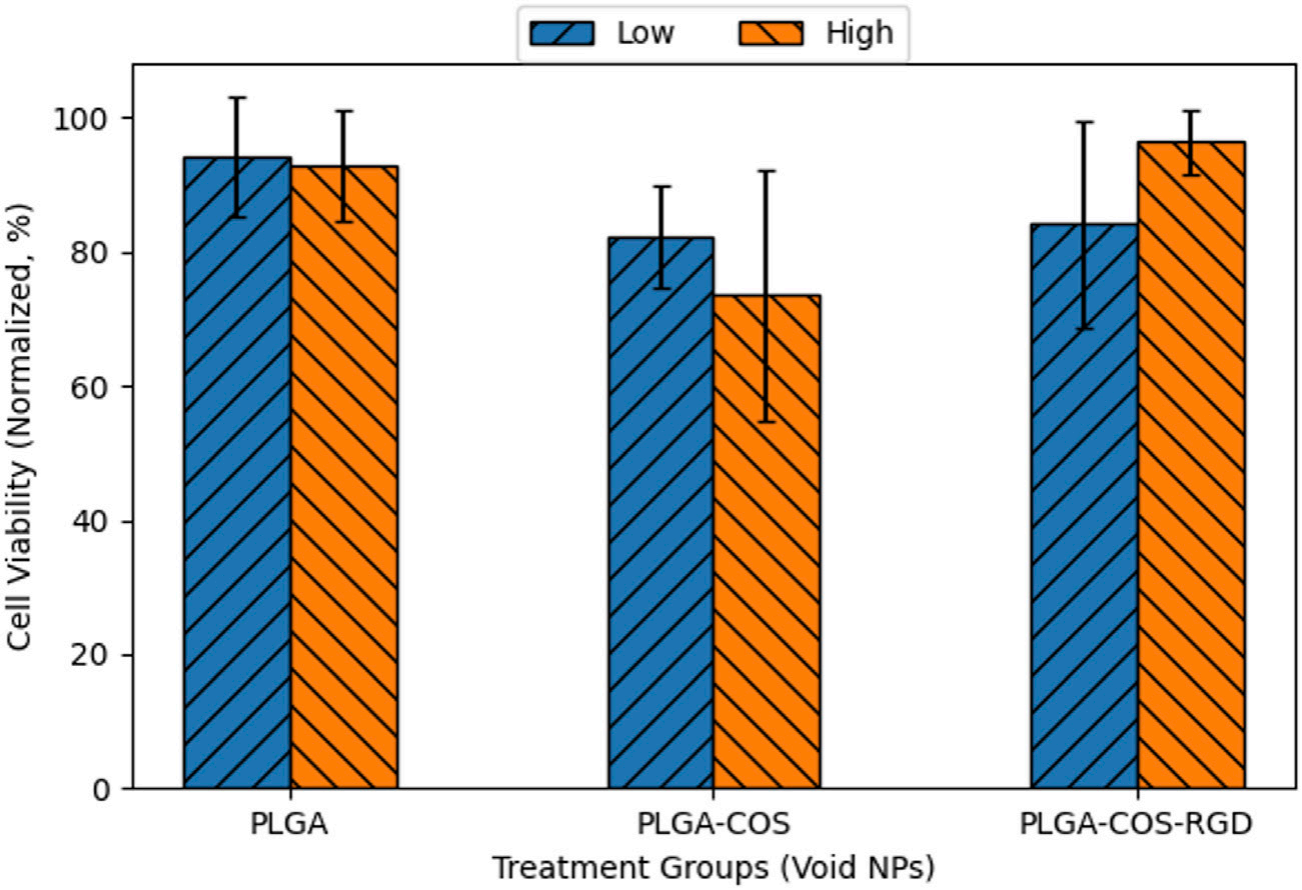
Normalized cell viability of 24 h treatment of OVCAR-3 cells with void NPs. Low and high concentrations of void NPs correspond to 5 and 40 μg/mL curcumin concentration if the NPs were loaded with curcumin. ANOVA (95% CI (α = 0.05), n = 3) found no significant difference between any of the sample groups.

To control for the effects of only curcumin and to rule out cytotoxic effects from the NP itself, OVCAR-3 cells were treated with void NPs over 24 h, shown in Figure 14. Each treatment group had a low and high treatment concentration, corresponding respectively to the lowest and highest concentration of curcumin-loaded NPs used in Figure 11. Statistical t-test analysis of the treatment groups did not find any statistically significant difference between high and low concentration samples. The void NPs themselves do not appear to contribute to the cytotoxicity of the curcumin treatment.

Together, these results, along with the fluorescent imaging of Coumarin-6 uptake indicates that, on its own, free curcumin is unable to affect cell viability, most likely due to its insolubility in aqueous medium. The NPs themselves do not appear to have cytotoxic effects, but are shown to be successfully uptaken by cells; when curcumin is encapsulated in these NPs, the curcumin is successfully uptaken by cells, facilitated by the NPs. Once in the cells, the curcumin exhibits cytotoxicity to the cancer cells.

Though cell studies overall did not show statistically significant differences between treatment groups of PLGA, PLGA-COS and PLGA-COS-RGD NPs, it is possible that an exploration into *in vitro* studies comparing healthy mammalian cells to tumour cells like OVCAR-3 could yield interesting results. The coating process requires further optimization, but the coating process may assist to some degree in avoiding uptake by healthy cells in addition to targeting tumour cells via their receptors.

## Discussion

In this study, curcumin was encapsulated in PLGA NPs. PLGA NPs were successfully synthesized through the emulsion method within acceptable size range (sub-500 nm diameter), and narrow distribution. EE% of curcumin was 60%, which was slightly lower than previous similar studies observed in literature; this may be due in part to minor degradation during processing and/or storage of the NPs. The NPs were successfully coated with COS, providing the nanocarrier with a positively charged surface. The surface was further modified by chemical addition of RGD peptide, though binding efficiency was less than 15%. Short-term stability study showed that the PLGA NPs and the COS coating were stable even when left in an aqueous solution over 48 h at ambient temperature.

The NP-encapsulated curcumin showed significant resistance to degradation in buffered aqueous conditions compared to free, unencapsulated curcumin. Under sink conditions, curcumin was rapidly released from the PLGA NPs, reaching 100% total release in less than 12 h. This runs counter to expectations from previous works with PLGA and curcumin in which those studies showed prolonged release over the course of several days.

Cell uptake studies using Coumarin-6 dye showed the PLGA NP ability to successfully deliver the encapsulated hydrophobic dye to OVCAR-3 cells, compared to unencapsulated Coumarin-6 control, which was unable to be uptaken by cells even after 24 h incubation. In cell toxicity studies, OVCAR-3 cancer cells treated with encapsulated curcumin showed significant toxicity to cells when compared to free curcumin. Void NP treatment was used to control for toxic effects from the NP material itself; cytotoxic effects from those samples were statistically insignificant. Free curcumin did not appear to show statistically significant cytotoxic effects even at concentrations where the encapsulated curcumin treatments experienced cell viability well below 50%. Overall, these results show the effectiveness of PLGA-based NPs in encapsulating, stabilizing, and delivering a hydrophobic, water-sensitive drug like curcumin. Lack of statistical differentiation between PLGA NPs coated with COS and RGD suggest those coating processes require further optimization. Further *in vitro* studies can be explored to compare uptake of NPs and cell viability after treatment by mammalian tumour cells vs. healthy mammalian cells.

Overall, the project was successful in synthesizing curcumin-encapsulating PLGA NPs using the emulsion method. The COS and RGD coatings showed some success, though further optimization is required. The functionalization of RGD to the amine-groups of COS was attempted under pH conditions favouring the conditions for EDC to react with the carboxyl groups on RGD; there may be more success in favouring pH conditions that would result in more deprotonation of the amine groups instead. Further studies are required to confirm whether the coatings are attached through chemical binding, or physical adsorption, particularly through more direct characterization techniques of the chemical composition of the NPs. This would include ^1^H NMR and FTIR. Cell studies showed that the NPs did vastly improve delivery of drug, but differences between PLGA, PLGA-COS and PLGA-COS-RGD NPs were inconclusive.

In addition to *in vitro* studies, *in vivo* studies might help determine how effectively the NPs can target tumours, and whether they will remain in the circulatory system for prolonged periods. *In vivo* studies may provide a more accurate assessment of whether the nanocarrier system can preferentially target tumour cells; with *in vitro* tumour cell studies, the cells likely uptake the NPs that are present in the treatment more quickly than healthy cells, and the well plate nature of the treatment means that the tumour cells likely absorb as much of the NPs as they require. On the other hand, with *in vivo* studies, the NPs will be circulating but at much lower overall concentration, which likely means that treatment will be more reliant on the rate of uptake. One way to measure effectiveness of treatment would be by measuring relative tumour sizes over a course of treatments [40]. A major challenge to *in vivo* studies will be tracking NP concentrations in the bloodstream, but also accumulation in tumour tissue vs. healthy tissue or potentially organs such as the kidneys/liver. While tracking drug in the bloodstream might utilise a similarly fluorescent dye in place of (or alongside) curcumin, accumulation of the NPs in different tissue could require a different solution. Manual biopsy and processing of tissue might be effective to determine concentrations in specific organs and obvious tumour tissue, but it may be less effective if the NPs/drugs are accumulating in other tissue like fat. Whole body fluorescence imaging has been used in mouse models to image distribution patterns of NP accumulation and could be used to determine whether NPs are accumulating in expected (or unexpected) locations in the body [41, 42].

Though research into NP-based solutions for drug delivery are becoming more popular, the field is still relatively new, and markets have not seen much widespread adoption of these technologies. It is hoped that this project will serve as a significant step towards the scalability and modularity that will enable the wider applicability and production of nanocarriers to enhance drug delivery and effectiveness. While this project focused on anti-cancer applications, there are numerous other potential areas of use, including anti-bacterial/anti-fungal agents, or delivery to areas of the body with otherwise low accumulation such as the past the blood-brain-barrier. Further optimization will be required to improve properties such as peptide binding; there may also be avenues to explore such as the use of click chemistry in chemical binding, as well as other polymers suited for applications like anti-bacterial activity.

## Data Availability

The raw data supporting the conclusions of this article will be made available by the authors, without undue reservation.
